# Potential role of p21 Activated Kinase 1 (PAK1) in the invasion and motility of oral cancer cells

**DOI:** 10.1186/s12885-016-2263-8

**Published:** 2016-05-16

**Authors:** Muraleedharan Parvathy, Sreeharshan Sreeja, Rakesh Kumar, Madhavan Radhakrishna Pillai

**Affiliations:** Cancer Research, Rajiv Gandhi Centre for Biotechnology, Thiruvananthapuram, Kerala India; Department of Biochemistry and Molecular Medicine, George Washington University, Washington DC, USA

**Keywords:** p21 Activated Kinase, Oral cancer, Invasion, Cytoskeletal remodelling, Migration

## Abstract

**Background:**

Oral cancer malignancy consists of uncontrolled division of cells primarily in and around the floor of the oral cavity, gingiva, oropharynx, lower lip and base of the tongue. According to GLOBOCAN 2012 report, oral cancer is one of the most common cancers among males and females in India. Even though significant advancements have been made in the field of oral cancer treatment modalities, the overall prognosis for the patients has not improved in the past few decades and hence, this demands a new thrust for the identification of novel therapeutic targets in oral cancer. p21 Activated Kinases (PAKs) are potential therapeutic targets that are involved in numerous physiological functions. PAKs are serine-threonine kinases and they serve as important regulators of cytoskeletal dynamics and cell motility, transcription through MAP kinase cascades, death and survival signalling, and cell-cycle progression. Although PAKs are known to play crucial roles in cancer progression, the role and clinical significance of PAKs in oral cancer remains poorly understood.

**Results:**

Our results suggest that PAK1 is over-expressed in oral cancer cell lines. Stimulation of Oral Squamous Cell Carcinoma (OSCC) cells with serum growth factors leads to PAK1 re-localization and might cause a profound cytoskeletal remodelling. PAK1 was also found to be involved in the invasion, migration and cytoskeletal remodelling of OSCC cells.

**Conclusions:**

Our study revealed that PAK1 may play a crucial role in the progression of OSCC. Studying the role of PAK1 and its substrates is likely to enhance our understanding of oral carcinogenesis and potential therapeutic value of PAKs in oral cancer.

**Electronic supplementary material:**

The online version of this article (doi:10.1186/s12885-016-2263-8) contains supplementary material, which is available to authorized users.

## Background

According to GLOBOCAN 2012 [1], there has been 14.1 million new cancer cases, 8.2 million cancer deaths and 32.6 million people living with cancer worldwide within 5 years of diagnosis. Carcinoma of lip and oral cavity is one of the most frequent causes of cancer deaths among males. Ninety percent of oral cancers are OSCC [2], originating from the flat cells (called squamous cells) that cover the surface of the oral cavity and oropharynx. With the advent of modern techniques, our understanding about the pathogenesis of oral cancer has increased over a period of time. But despite this, oral cancer has one of the lowest survival rates- 50 percent, within a five-year period (http://www.oralcancerfoundation.org/facts/). This calls for the identification of new therapeutic targets and one such potential set of target molecules are p21 Activated Kinases (PAKs).

PAKs were one of the first classes of Rho-GTPases-regulated kinases [3] to be identified. PAKs are a family of evolutionarily conserved group of serine-threonine kinases. So far, six PAK family members have been identified in mammals [4] and they have been classified into two sub-groups- Group I PAKs (PAKs 1-3) and Group II PAKs (PAKs 4-6). While Group I PAKs are activated by a variety of GTPase -dependent and -independent mechanisms, Group II PAKs are constitutively activated. PAKs are considered prime regulators of the actin cytoskeleton and motility [5]. Due to their central role in actin remodelling and their ability to activate Matrix metalloproteinases (MMPs), Rho GTPases play an important role in tumor cell invasion and metastasis [6]. The current evidence suggests the involvement of PAKs in motility [7–10], cell survival [11, 12], anchorage-independent growth [13], angiogenesis [14–18], invasion [18, 19], migration [20, 21] and regulation of cell cycle and mitosis [22, 23]. Consequently, PAKs have also been implicated in a number of pathological conditions including cancer [24].

Our interest resides in the PAK1 kinase, a member of group I PAKs. The roles of PAK1 in breast [25–30], colon [31–33], lung [34, 35], melanoma [36], prostate [37, 38], ovarian cancers [39] are well studied. Even though PAKs are known to play roles in the crucial steps of cancer progression, its role and clinical significance in oral cancer remains ill defined. In this work, we investigated the role of PAK1 in oral cancer. Our study suggests that PAK1 plays a crucial role in the progression of oral cancer and represents a potential therapeutic target in OSCC.

## Results and Discussion

### OSCC cell lines showed differential expression and localization patterns for PAK1

**Fig. 1:**
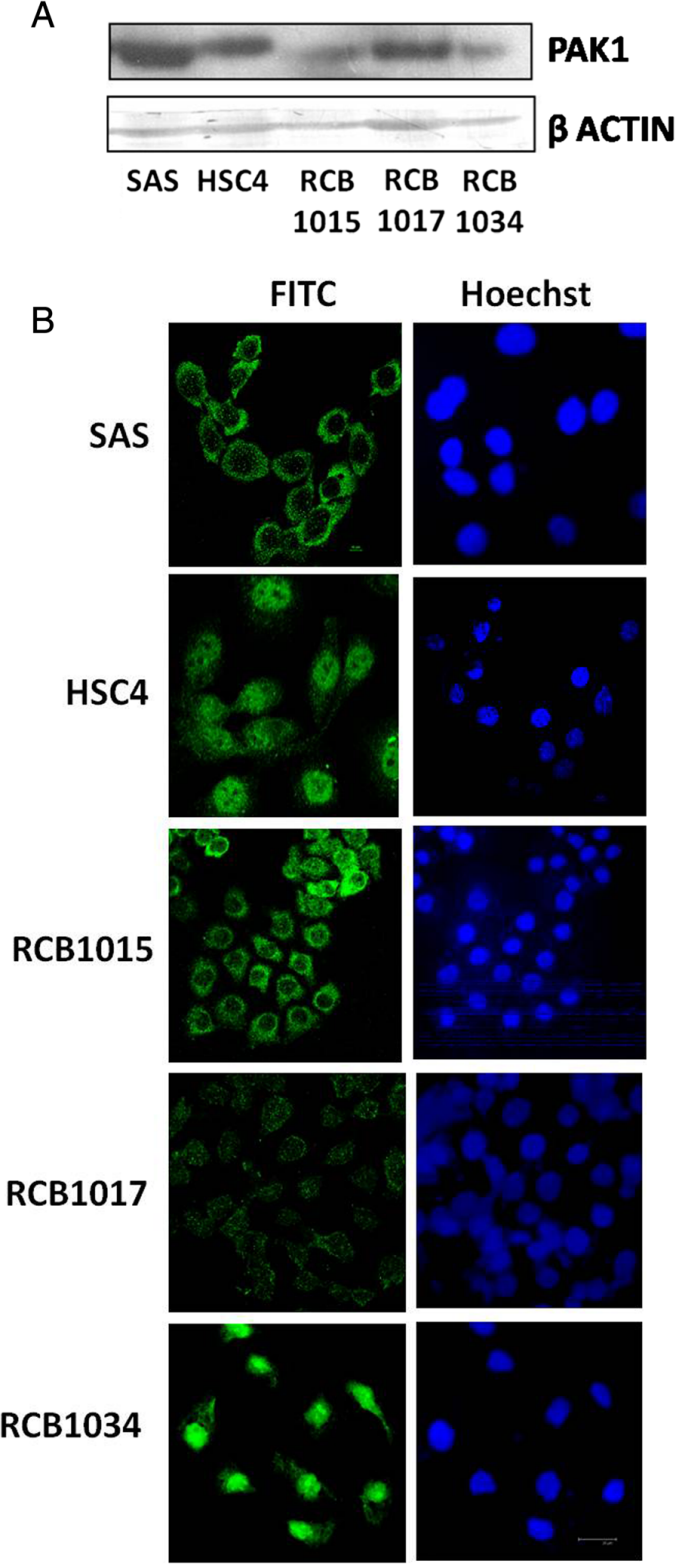
Expression and localization patterns of PAK1 in the five OSCC cell lines. **a** OSCC cell lines (SAS, HSC4, RCB1015, RCB1017 and RCB1034) used in our study showed differential expression levels of PAK1. **b** PAK1 exhibited differential localization patterns in OSCC cell lines. PAK1 predominantly localized in the nucleus in HSC4 cells, whereas in SAS and RCB1015, PAK1 showed a predominant cytoplasmic localization. While in RCB1017, PAK1 was found in both nuclear and cytoplasmic compartments, in RCB1034, PAK1 was mostly showing nuclear localization pattern

Very few studies have elucidated the expression pattern of PAK1 in OSCC cell lines. We analysed the expression and localization patterns of PAK1 in the five OSCC cell lines. Our study demonstrated that all the five OSCC cell lines differentially expressed PAK1 (Fig. 1a).

Localization pattern of proteins play a key role in cancer. Previous studies have shown that PAK1 localizes in the cytoplasm, nucleus or to the cell membrane. An earlier study had demonstrated that PAK1 expression in breast tumors positively correlated with tumor grade, with higher expression in grade 3 [40]. Another study involving an established murine model of breast cancer progression had shown that PAK1 expression and its nuclear localization were progressively increased during the transition from ductal hyperplasia to ductal carcinoma *in situ* to adenocarcinoma [41]. Holm et al. [42] had suggested that PAK1 activation and its nuclear localization may be one of the mechanisms responsible for reduced tamoxifen sensitivity of breast tumor cells. Even though there are studies in other cancer types that correlated the localization patterns and cancer progression, this issue has not been investigated in oral cancer as yet.

To study the localization patterns of PAK1 in OSCC cell lines, confocal microscopy was performed in OSCC cell lines which were exponentially growing in 10 % Foetal Bovine Serum containing medium. The results of confocal microscopy revealed that PAK1 predominantly localizes in the nucleus of HSC4 cells and RCB1034 cells, while in SAS cells and RCB1015, PAK1 was predominantly in the cytoplasm (Fig. 1b). In the case of RCB1017, PAK1 was observed in both the nuclear and cytoplasmic compartments.

### Differential localization of PAK1 and cytoskeleton remodelling in serum-stimulated OSCC cells

Growth factors and their receptors play a very crucial role in cancer progression. Previous reports suggested that over-expression of Epidermal Growth Factor Receptor (EGFR) [43] and Transforming Growth Factor β1 (TGF β1) [44] are associated with increased malignant potential and correlated with poor treatment outcome in head and neck cancer. Growth factors are known to recruit PAKs to the membranes, where they come in contact with other activating kinases, leading to the downstream signalling cascade.

**Fig. 2:**
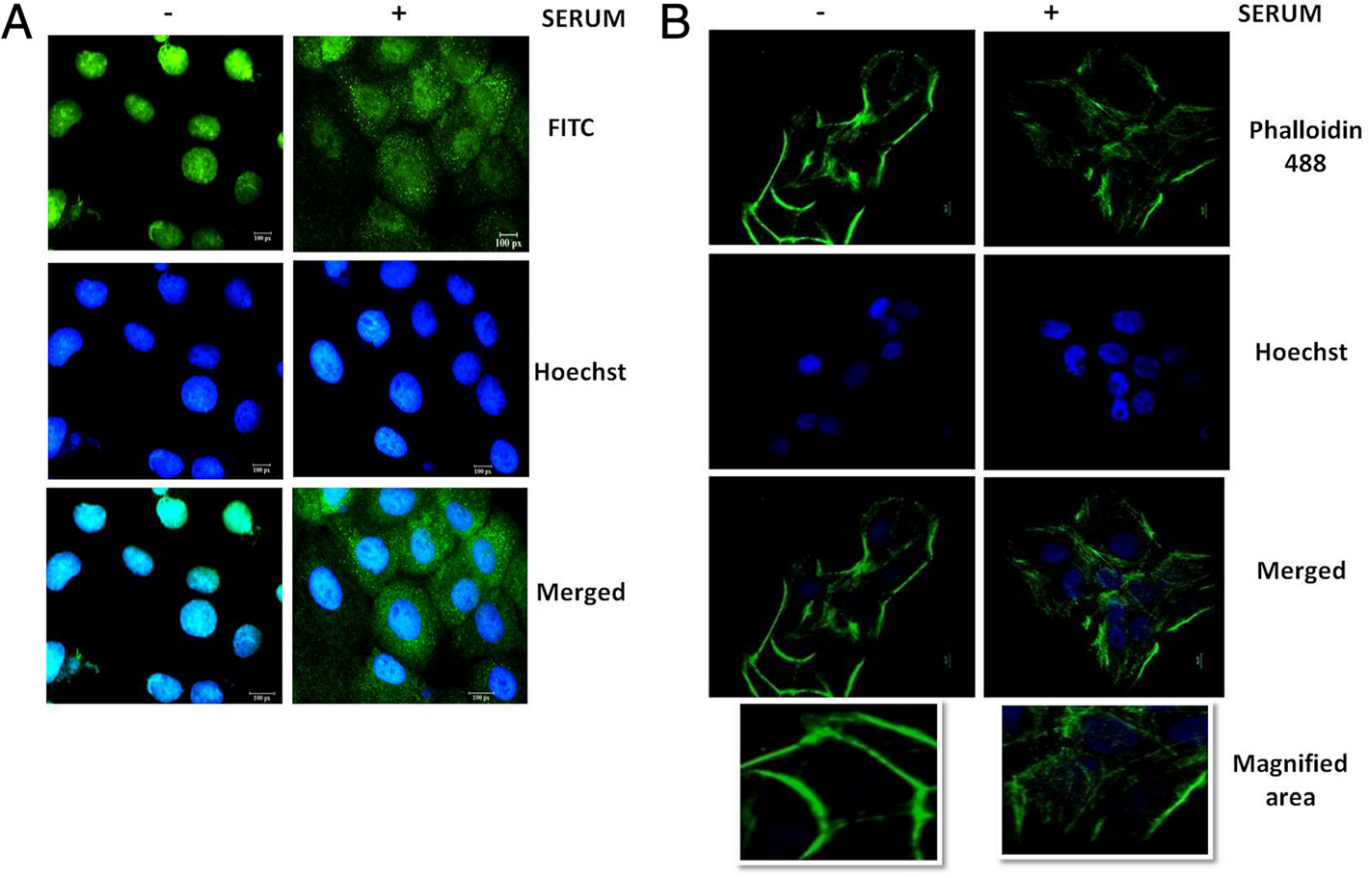
Effect of serum growth factors on PAK1 localization and its potential role in regulating actin cytoskeletal structures. HSC4 cells were serum starved for 48 h and treated with or without medium containing 10 % FBS for varying intervals of time. One set of plates were subjected to localization studies (**a**) while the second set was stained with Phalloidin 488 (**b**)

To study the effect of serum growth factors on the localization of PAK1, HSC4 cells were grown in serum free condition. After 48 h, cells were cultured in the presence of medium containing 10 % serum for varying time intervals and the localization patterns of PAK1 were studied. It was observed that in serum-free condition, PAK1 predominantly resides in the nucleus, but upon stimulation with serum, PAK1 largely accumulates in the cytoplasm (Fig. 2a). This suggests that PAK1 localization is regulated by the growth factors and in-turn, differential sub-cellular PAK1 localization may influence its ability to trigger cytoskeleton remodelling in the cytoplasm.

To analyse the effect of growth factors on actin remodelling in OSCC, HSC4 cells were grown in serum free condition. After 48 h, cells were cultured in medium containing 10 % serum for varied time intervals and Phalloidin staining was done after fixing the cells. As shown in Fig. 2b, actin was mostly in the condensed form and was concentrated mostly in the periphery of the cells grown in serum-free conditions. However, a more prominent actin network was visible in serum-stimulated cells. These observations suggest that PAK1 predominantly localizes in the nucleus in serum-starved OSCC cells with a condensed actin network. However, PAK1 translocates to the cytoplasm in serum-stimulated cells wherein it might participate in actin remodelling. Further experiments would provide conclusive evidence regarding the suggested role of PAK1 in regulating the cytoskeletal structures of OSCC cells.

### Potential role of PAK1 in the cytoskeletal remodelling, invasiveness and motility of OSCC cells

**Fig. 3:**
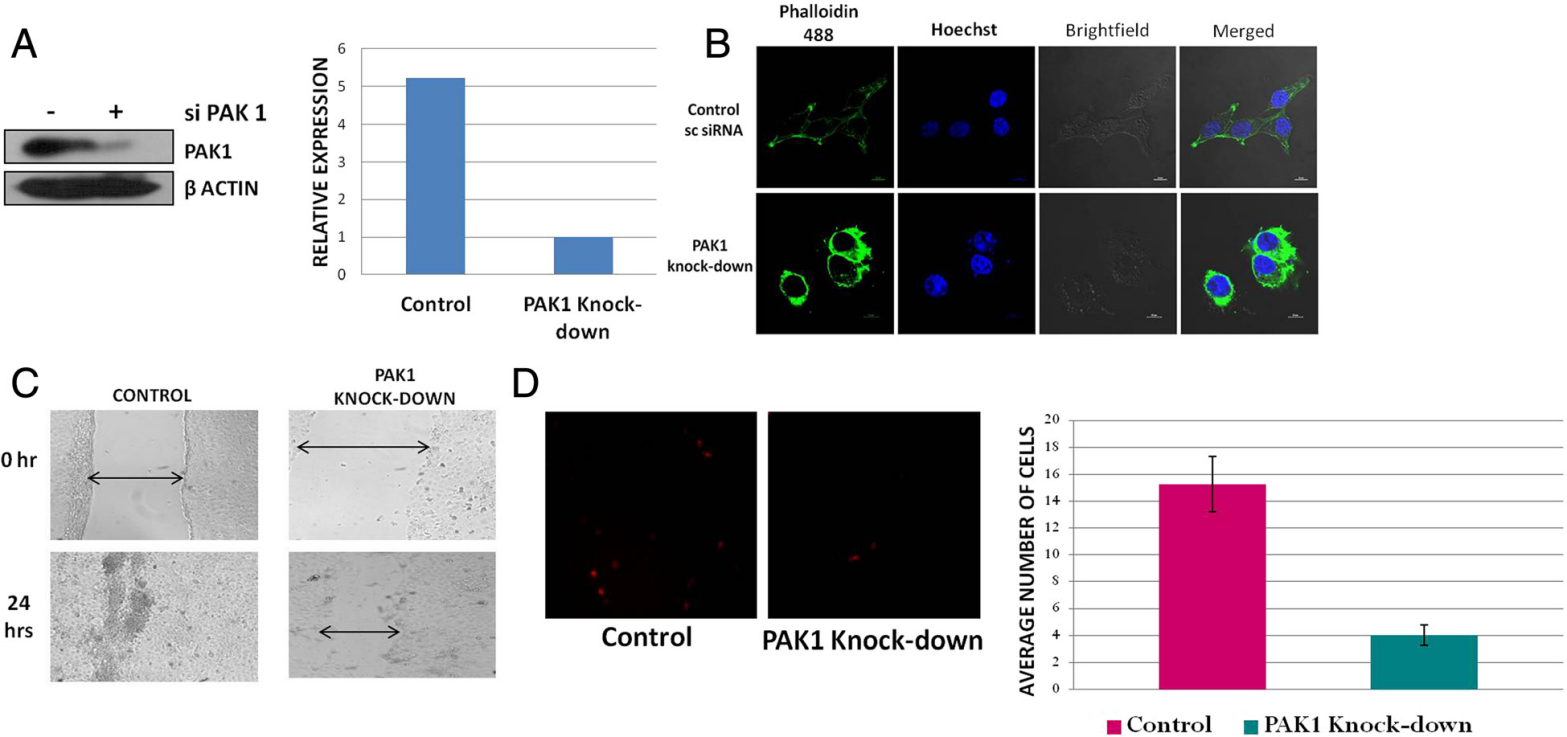
Biological effects of PAK1 knock-down in OSCC cells. **a** Immunoblot analysis of PAK1 expression in control and PAK1 knock-down SAS cells and its densitometric quantitation. **b** Phalloidin staining showing actin structures in the SAS cells treated with the control or PAK1 siRNA. **c** Wound healing assay in the SAS cells treated with the control or PAK1 siRNAs. **d** Fluorescent images obtained after matrigel invasion assay, showing the cells (stained with Dil C) that have migrated through the matrigel. A quantitative representation of the image data is also illustrated

Cytoskeletal remodelling plays an essential role in the cell motility. For a productive cancer progression, the malignant cells must undergo dynamic changes in cytoskeletal structure, thereby enabling the cancer cells to migrate and invade the neighbouring tissues. As mentioned earlier, PAK1 has been implicated in the progression of different cancer types. However, its role in oral cancer remains poorly studied. To study the effect of PAK1 in the cytoskeletal remodelling, motility and invasiveness of OSCC cells, PAK1 was selectively knocked down in the SAS cells (Fig. 3a) and such cells were subjected to Phalloidin staining, wound healing assay and Matrigel invasion assay. Phalloidin staining of control and PAK1 kock-down SAS cells **(**Fig. 3b**)** demonstrated that the actin structures and the morphology of the cells were intact in the control cells with PAK1, while these structures were profoundly reorganized in OSCC cells with depleted PAK1, indicating that PAK1 might be essential for cytoskeletal remodelling of OSCC cells. Further experiments are required to establish the role of PAK1 in cytoskeletal remodelling of OSCC cells.

To study the motility of OSCC cells with or without PAK1 knock-down, we next performed Wound healing assay. It was observed that even after 24 h of growing the cells with PAK1 knock-down in serum containing medium, the wound was not completely healed (Fig. 3c) as compared to the control cells with almost a complete wound healing. To exclude a potential influence of expected proliferation difference in 10 % FBS-containing medium, a set of cells were also grown in growth factor-free medium. This observation suggested that PAK1 may be involved in the motility of growth-factor stimulated OSCC cells.

Another pre-requisite for cancer progression is the ability of cancer cells to invade into the neighbouring normal tissue, thereby facilitating the process of metastasis. To study the involvement of PAK1 in this process, we subjected OSCC cells with or without PAK1 knock-down to Matrigel invasion assay. It was observed that when compared to the control cells, cells with PAK1 knock-down showed almost four fold decrease in the invasiveness, suggesting that PAK1 is also involved in the invasiveness of OSCC cells (Fig. 3d).

Additionally, we had done an *in-silico* analysis to survey PAK1 phosphorylation sites in differentially expressed genes in OSCC by using the software GPS (Group-based prediction system) 2.1 [45]. Out of the 64 genes that are differentially expressed in OSCC, 26 genes (Additional file 1) were found to have PAK1 phosphorylation sites. Further functional studies are required to confirm this *in-silico* finding.

## Conclusions

We had ventured into an under-investigated area in cancer biology i.e., the role of PAK1 in the progression of OSCC. PAK1 was found to be differentially expressed and localized in OSCC cell lines used for our study. Because growth factors are potent inducers of PAK1 activation, we studied the response of OSCC cells by serum growth factors. It was observed that serum growth factors facilitate the translocation and/or accumulation of PAK1 in distinct sub-cellular compartments, and also, it might affect the cytoskeletal structures of OSCC cells. Migration and invasion of cancer cells are under the control of dynamic cytoskeletal re-organization, in addition to other pathways. We found that PAK1 plays a potential role in cytoskeletal remodelling of OSCC cells, thereby affecting the motility and invasiveness of OSCC cells. Further studies are required to bring out the mechanistic insights into the role of PAK1 in oral cancer progression. In brief, PAK1 plays a crucial role in OSCC progression and that studying the role of PAK1 could improve our understanding of oral carcinogenesis and in determining new therapeutic targets for oral cancer.

## Methods

### Cell culture

Oral cancer cell lines like SAS, HSC4, RCB 1015, RCB1017 and RCB1034 were used for the study. Out of this, SAS and HSC4 are human tongue squamous cell carcinoma cell line whereas RCB1015, RCB1017 and RCB1034 are OSCC cell line obtained from the same patient in different stages of tumor progression. RCB1015 is a G-CSF producing OSCC cell line, RCB1017 is a G-CSF and IL-1 producing oral squamous cell carcinoma whereas, RCB1034 is a human cell line derived from metastasis of cancer occurred in oral cavity. All the cell lines were obtained from Riken BRC (BioResource Center) Cell bank, Japan. The cells were grown in Dulbecco’s modified Eagles medium (DMEM) (Sigma-Aldrich, St. Louis, MO) and RPMI 1640 medium (developed at Roswell Park Memorial Institute) supplemented with 10 % heat-inactivated fetal bovine serum (FBS; Invitrogen), and antibiotic and anti-mycotic cocktail (Lonza).

### Immunoblot analysis

Immunoblot analysis was done as mentioned in Haneef et al. [46]. Briefly, cells were washed with PBS and lysed using RIPA (Radioimmunoprecipitation assay) buffer (150 mM NaCl, 1 % NP-40, 0.5 % Sodium deoxycholate, 0.1 % SDS, 50 mM Tris–HCl pH 7.4) containing protease inhibitor cocktail (Sigma-Aldrich Inc., USA). Briefly, equal amount of protein as determined by Bradford assay was subjected to SDS-PAGE, followed by transfer to nitrocellulose membrane (Millipore, MA). The membrane was then blocked in 5 % powdered non-fat milk Tris solution for 1 h. Membrane was then incubated overnight with primary antibody, followed by incubation with species-specific horseradish peroxidase (HRP) conjugated secondary antibody (1:5000, Santa Cruz) at room temperature for 1 h. Protein bands were visualised on X-ray film using ECL-plus reagents (Amersham, NJ). The primary antibodies were: PAK1 (1:5000, Bethyl Labs) and Beta actin (1:5000, Sigma).

### Confocal immunofluorescent microscopy and phalloidin staining

The oral cell lines (SAS, HSC4, RCB1015, RCB1017 and RCB1034) were cultured on glass coverslips until 60–70 % confluence. Cells were fixed in chilled 4 % paraformaldehyde solution in PBS at 4 °C for 7 min and then permeabilized with 0.1 % Triton X 100 for 7 min at room temperature, followed by three washes using PBS. Non-specific protein binding was reduced by incubation in 3 % BSA at room temperature for 1 h. The cells were then incubated with primary antibodies for PAK1 (dilution: 1:500) at 4 °C for overnight. The cells were washed twice with PBS, followed by incubation with secondary antibody conjugate for 1 h at room temperature and nuclear staining was done using Hoechst staining (5 μg/ml) for 5 min. For Phalloidin staining, the cells were treated with Alexa Fluor® 488 Phalloidin (dilution: 5 μM) for 20 min. After giving a wash with PBS twice, the cells were stained with the nuclear dye Hoechst 33342 (Invitrogen, 5 μg/ml). Again, the cells were washed twice in PBS. The coverslips were mounted in Glycerol on glass slides and sealed with nail polish. Microscopic observations were made using laser scanning confocal microscope (Nikon A1R). Localization pattern of PAK1 was studied using Immunofluorescent detection of the protein while F-Actin structure was studied using Phalloidin staining.

### siRNA transfection

SAS cells were seeded on 6 well plates and incubated for 48 h in complete medium. The cells were transfected with 100nM of siRNA specific for human PAK1 (pSUPER PAK1 siRNA) or control siRNA (sc-37007, Santacruz) using the Lipofectamine LTX and PLUS siRNA transfection reagents as per the manufacturer’s instruction. 12 h after transfection, transfection medium was replaced with fresh medium containing serum and allowed to grow for 24 h. Whole cell extract was prepared 36 h after transfection for analysis of silencing efficiency by western blot using antibody against PAK1 (Bethyl Labs, A301-259A).

### Matrigel invasion assay

The cell invasion assay was conducted using BioCoat Matrigel (Becton Dickinson Biosciences) by following the manufacturer’s protocol. After transfection with either anti-PAK1 or control siRNAs, cells were seeded into the upper chamber at a density of 1 × 10^5^ cells per well in 0.2 ml of 1 % FBS medium. The lower chamber contained complete culture medium, which included 10 % FBS to attract invading cells. Cells were incubated at 37 °C for 24 h, and the number of cells that invaded through the Matrigel-coated membranes was counted and compared with the number of cells that passed through the membrane in the control chambers. The invaded cells on lower side of membrane were fixed, stained with Dil C stain, and photographed.

### Wound healing assay

Cell migration was evaluated by an *in vitro* wound-healing assay. After transfection with either anti-PAK1 or scramble siRNAs, 5 × 10^5^ cell/ml transfectants was seeded in a 24 well plate. After the culture had formed a monolayer, a cell-free gap was produced with the help of a yellow tip. After changing to culture medium with 1 % FBS, the cell migration status toward the gap area was photographed after 24 h.

## Additional file

Additional file 1: The FASTA sequences of differentially expressed genes were examined using GPS2.1 software to analyse the presence of PAK1 phosphorylation sites. Using highly stringent condition, we recognized 26 genes with putative PAK1 phosphorylation sites. (XLSX 10 kb)

## Abbreviations

DMEM: Dulbecco’s modified eagles medium
EGFR: Epidermal Growth Factor Receptor
FBS: Foetal Bovine Serum
GPS2.1: Group-based prediction system 2.1
GTPases: Guanosine TriPhosphatases
HRP: Horseradish peroxidase
KD: knock-down
mM: milli molar
MMPs: matrix metalloproteinases
OSCC: Oral Squamous Cell Carcinoma
PAK1: p21 Activated Kinase 1
PBS: phosphate buffered saline
RIPA: Radioimmunoprecipitation assay
TGF β1: Transforming Growth Factor β1
μg/ml: microgram per milliliter
